# Methyl 6-bromo-7-meth­oxy-1,3-benzodioxole-5-carboxyl­ate

**DOI:** 10.1107/S1600536808019399

**Published:** 2008-07-05

**Authors:** Xianshu Fu, Zihong Ye, Lifei Mao, Sasa Shao

**Affiliations:** a College of Life Science, China Jiliang University, Hangzhou 310018, People’s Republic of China

## Abstract

The non-H atoms of the title compound, C_10_H_9_BrO_5_, are essentially coplanar, with the exception of the ester group [the O=C—O—C torsion angle is −143.4 (3)°].

## Related literature

For related literature, see: Gerhard *et al.* (2003 ▶); Song & Xiao (1982 ▶).
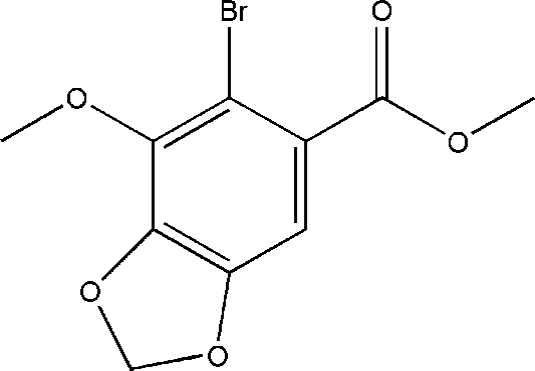


## Experimental

### 

#### Crystal data

C_10_H_9_BrO_5_
*M* *_r_* = 289.08
Triclinic, 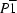

*a* = 7.6933 (12) Å
*b* = 8.0616 (13) Å
*c* = 9.7039 (15) Å
α = 105.062 (2)°
β = 91.667 (2)°
γ = 113.457 (2)°
*V* = 527.13 (14) Å^3^
*Z* = 2
Mo *K*α radiation
μ = 3.90 mm^−1^
*T* = 294 (2) K
0.22 × 0.18 × 0.08 mm

#### Data collection

Bruker SMART CCD area-detector diffractometer
Absorption correction: multi-scan (*SADABS*; Bruker, 1997 ▶) *T*_min_ = 0.481, *T*_max_ = 0.745
2997 measured reflections
2129 independent reflections
1790 reflections with *I* > 2σ(*I*)
*R*_int_ = 0.017

#### Refinement

*R*[*F* ^2^ > 2σ(*F* ^2^)] = 0.035
*wR*(*F* ^2^) = 0.096
*S* = 1.03
2129 reflections
147 parameters
H-atom parameters constrained
Δρ_max_ = 0.67 e Å^−3^
Δρ_min_ = −0.42 e Å^−3^

### 

Data collection: *SMART* (Bruker, 1997 ▶); cell refinement: *SAINT* (Bruker, 1997 ▶); data reduction: *SAINT*; program(s) used to solve structure: *SHELXS97* (Sheldrick, 2008 ▶); program(s) used to refine structure: *SHELXL97* (Sheldrick, 2008 ▶); molecular graphics: *SHELXTL* (Sheldrick, 2008 ▶); software used to prepare material for publication: *SHELXTL*.

## Supplementary Material

Crystal structure: contains datablocks global, I. DOI: 10.1107/S1600536808019399/tk2272sup1.cif

Structure factors: contains datablocks I. DOI: 10.1107/S1600536808019399/tk2272Isup2.hkl

Additional supplementary materials:  crystallographic information; 3D view; checkCIF report

## Figures and Tables

**Fig. 1.**
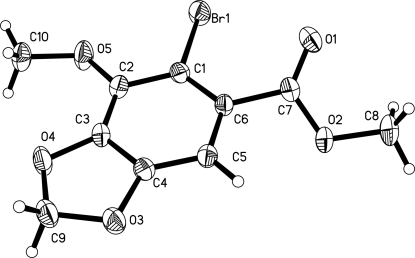
View of (I) showing the atom-labelling scheme. Displacement ellipsoids are drawn at the 35% probability level.

## supplementary crystallographic information

### Comment

The title compound, (I), is a key intermediate for the preparation of biphenyl derivatives. These may act to moderate liver ailments and, thus, be effective in the treatment of acute and chronic hepatitis (Song & Xiao, 1982). The non-hydrogen atoms in (I), Fig. 1, are essentially co-planar with the exception of the ester group; the O1-C7-C6-C1 torsion angle is -143.4 (3)°.

### Experimental

The title compound (I) was prepared according to the procedure of Gerhard *et al.* (2003). The reaction was initiated by the addition of one molar equivalent of methanol to one molar equivalent of 6-bromo-7-methoxybenzo[*d*][1,3]dioxole-5-carboxylic acid in dichloromethane solution and subsequent stirring at room temperature for 12 h. A white powder resulted (yield 88%) and single crystals suitable for X-ray analysis were obtained by slow evaporation of an acetonitrile solution; m.p. 357 K.

### Refinement

All H atoms were positioned geometrically and refined in the riding approximation with C—H = 0.93–0.97 Å, and with *U*_iso_(H) = 1.2*U*_eq_(carrier atom) and 1.5*U*_eq_(methyl-C).

### Crystal data

**Table:**

C_10_H_9_BrO_5_	*Z* = 2
*M**_r_* = 289.08	*F*_000_ = 288
Triclinic, *P*1	*D*_x_ = 1.821 Mg m^−^^3^
Hall symbol: -P 1	Melting point: 357 K
*a* = 7.6933 (12) Å	Mo *K*α radiation λ = 0.71073 Å
*b* = 8.0616 (13) Å	Cell parameters from 1718 reflections
*c* = 9.7039 (15) Å	θ = 2.9–26.3º
α = 105.062 (2)º	µ = 3.90 mm^−^^1^
β = 91.667 (2)º	*T* = 294 (2) K
γ = 113.457 (2)º	Block, colorless
*V* = 527.13 (14) Å^3^	0.22 × 0.18 × 0.08 mm

### Data collection

**Table:**

Bruker SMART CCD area-detector diffractometer	2129 independent reflections
Radiation source: fine-focus sealed tube	1790 reflections with *I* > 2σ(*I*)
Monochromator: graphite	*R*_int_ = 0.017
*T* = 294(2) K	θ_max_ = 26.4º
φ and ω scans	θ_min_ = 2.2º
Absorption correction: multi-scan(SADABS; Bruker, 1997)	*h* = −9→9
*T*_min_ = 0.481, *T*_max_ = 0.745	*k* = −7→10
2997 measured reflections	*l* = −11→12

### Refinement

**Table:**

Refinement on *F*^2^	Secondary atom site location: difference Fourier map
Least-squares matrix: full	Hydrogen site location: inferred from neighbouring sites
*R*[*F*^2^ > 2σ(*F*^2^)] = 0.035	H-atom parameters constrained
*wR*(*F*^2^) = 0.096	*w* = 1/[σ^2^(*F*_o_^2^) + (0.0596*P*)^2^ + 0.1433*P*] where *P* = (*F*_o_^2^ + 2*F*_c_^2^)/3
*S* = 1.03	(Δ/σ)_max_ = 0.001
2129 reflections	Δρ_max_ = 0.67 e Å^−^^3^
147 parameters	Δρ_min_ = −0.42 e Å^−^^3^
Primary atom site location: structure-invariant direct methods	Extinction correction: none

### Special details

**Table:**

Geometry. All e.s.d.'s (except the e.s.d. in the dihedral angle between two l.s. planes) are estimated using the full covariance matrix. The cell e.s.d.'s are taken into account individually in the estimation of e.s.d.'s in distances, angles and torsion angles; correlations between e.s.d.'s in cell parameters are only used when they are defined by crystal symmetry. An approximate (isotropic) treatment of cell e.s.d.'s is used for estimating e.s.d.'s involving l.s. planes.
Refinement. Refinement of *F*^2^ against ALL reflections. The weighted *R*-factor *wR* and goodness of fit *S* are based on *F*^2^, conventional *R*-factors *R* are based on *F*, with *F* set to zero for negative *F*^2^. The threshold expression of *F*^2^ > σ(*F*^2^) is used only for calculating *R*-factors(gt) *etc*. and is not relevant to the choice of reflections for refinement. *R*-factors based on *F*^2^ are statistically about twice as large as those based on *F*, and *R*- factors based on ALL data will be even larger.

### Fractional atomic coordinates and isotropic or equivalent isotropic displacement parameters (Å^2^)

**Table:**

	*x*	*y*	*z*	*U*_iso_*/*U*_eq_	
Br1	0.35252 (5)	1.09757 (4)	0.17325 (3)	0.05585 (16)	
O1	0.4065 (4)	0.7325 (4)	0.0679 (3)	0.0741 (8)	
O2	0.1696 (3)	0.4868 (3)	0.1074 (2)	0.0508 (5)	
O3	0.1494 (4)	0.7239 (3)	0.6385 (2)	0.0559 (6)	
O4	0.1874 (4)	1.0334 (3)	0.6889 (2)	0.0591 (6)	
O5	0.2912 (4)	1.2488 (3)	0.4655 (2)	0.0627 (7)	
C1	0.2920 (4)	0.9669 (4)	0.3141 (3)	0.0379 (6)	
C2	0.2692 (4)	1.0680 (4)	0.4491 (3)	0.0411 (6)	
C3	0.2222 (4)	0.9728 (4)	0.5508 (3)	0.0393 (6)	
C4	0.1986 (4)	0.7872 (4)	0.5206 (3)	0.0394 (6)	
C5	0.2151 (4)	0.6849 (4)	0.3893 (3)	0.0399 (6)	
H5	0.1945	0.5589	0.3711	0.048*	
C6	0.2645 (4)	0.7784 (4)	0.2832 (3)	0.0362 (6)	
C7	0.2905 (4)	0.6697 (4)	0.1411 (3)	0.0430 (6)	
C8	0.1837 (6)	0.3669 (5)	−0.0273 (4)	0.0601 (9)	
H8A	0.1717	0.4182	−0.1041	0.090*	
H8B	0.0831	0.2419	−0.0467	0.090*	
H8C	0.3057	0.3608	−0.0206	0.090*	
C9	0.1525 (6)	0.8809 (5)	0.7482 (3)	0.0559 (8)	
H9A	0.2526	0.9183	0.8279	0.067*	
H9B	0.0307	0.8472	0.7844	0.067*	
C10	0.3163 (6)	1.3726 (5)	0.6061 (4)	0.0565 (8)	
H10A	0.1953	1.3405	0.6412	0.085*	
H10B	0.3653	1.5008	0.6025	0.085*	
H10C	0.4053	1.3601	0.6697	0.085*	

### Atomic displacement parameters (Å^2^)

**Table:**

	*U*^11^	*U*^22^	*U*^33^	*U*^12^	*U*^13^	*U*^23^
Br1	0.0810 (3)	0.0505 (2)	0.0386 (2)	0.02651 (18)	0.01453 (16)	0.01850 (15)
O1	0.0930 (19)	0.0607 (15)	0.0638 (17)	0.0274 (14)	0.0469 (15)	0.0149 (13)
O2	0.0636 (13)	0.0423 (11)	0.0361 (11)	0.0205 (10)	0.0119 (10)	−0.0025 (9)
O3	0.0856 (16)	0.0481 (12)	0.0368 (11)	0.0277 (12)	0.0195 (11)	0.0165 (10)
O4	0.0962 (18)	0.0519 (13)	0.0328 (11)	0.0369 (13)	0.0203 (11)	0.0079 (10)
O5	0.111 (2)	0.0402 (12)	0.0420 (12)	0.0379 (13)	0.0153 (13)	0.0095 (10)
C1	0.0412 (14)	0.0408 (15)	0.0303 (13)	0.0155 (12)	0.0058 (11)	0.0109 (11)
C2	0.0475 (16)	0.0350 (14)	0.0372 (15)	0.0179 (12)	0.0028 (12)	0.0045 (12)
C3	0.0452 (15)	0.0398 (14)	0.0293 (13)	0.0189 (12)	0.0044 (11)	0.0031 (11)
C4	0.0432 (15)	0.0418 (15)	0.0321 (14)	0.0166 (12)	0.0060 (11)	0.0110 (12)
C5	0.0465 (15)	0.0353 (14)	0.0370 (15)	0.0181 (12)	0.0078 (12)	0.0073 (11)
C6	0.0384 (14)	0.0353 (14)	0.0309 (13)	0.0152 (11)	0.0050 (11)	0.0040 (11)
C7	0.0495 (16)	0.0460 (16)	0.0348 (15)	0.0239 (14)	0.0099 (13)	0.0073 (13)
C8	0.076 (2)	0.058 (2)	0.0400 (17)	0.0346 (18)	0.0086 (16)	−0.0053 (15)
C9	0.076 (2)	0.059 (2)	0.0340 (16)	0.0299 (17)	0.0155 (15)	0.0121 (14)
C10	0.080 (2)	0.0455 (17)	0.0461 (18)	0.0349 (17)	0.0083 (16)	0.0027 (14)

### Geometric parameters (Å, °)

**Table:**

Br1—C1	1.895 (3)	C3—C4	1.382 (4)
O1—C7	1.192 (4)	C4—C5	1.366 (4)
O2—C7	1.336 (4)	C5—C6	1.404 (4)
O2—C8	1.446 (4)	C5—H5	0.9300
O3—C4	1.375 (3)	C6—C7	1.496 (4)
O3—C9	1.418 (4)	C8—H8A	0.9600
O4—C3	1.375 (3)	C8—H8B	0.9600
O4—C9	1.424 (4)	C8—H8C	0.9600
O5—C2	1.363 (3)	C9—H9A	0.9700
O5—C10	1.421 (4)	C9—H9B	0.9700
C1—C6	1.396 (4)	C10—H10A	0.9600
C1—C2	1.408 (4)	C10—H10B	0.9600
C2—C3	1.374 (4)	C10—H10C	0.9600
			
C7—O2—C8	115.8 (3)	O1—C7—O2	123.3 (3)
C4—O3—C9	105.9 (2)	O1—C7—C6	125.9 (3)
C3—O4—C9	105.8 (2)	O2—C7—C6	110.8 (2)
C2—O5—C10	119.7 (2)	O2—C8—H8A	109.5
C6—C1—C2	122.1 (3)	O2—C8—H8B	109.5
C6—C1—Br1	121.5 (2)	H8A—C8—H8B	109.5
C2—C1—Br1	116.4 (2)	O2—C8—H8C	109.5
O5—C2—C3	126.2 (3)	H8A—C8—H8C	109.5
O5—C2—C1	117.2 (3)	H8B—C8—H8C	109.5
C3—C2—C1	116.6 (2)	O3—C9—O4	108.6 (2)
C2—C3—O4	129.3 (3)	O3—C9—H9A	110.0
C2—C3—C4	121.1 (2)	O4—C9—H9A	110.0
O4—C3—C4	109.6 (2)	O3—C9—H9B	110.0
C5—C4—O3	126.8 (3)	O4—C9—H9B	110.0
C5—C4—C3	123.3 (3)	H9A—C9—H9B	108.3
O3—C4—C3	109.8 (2)	O5—C10—H10A	109.5
C4—C5—C6	117.0 (3)	O5—C10—H10B	109.5
C4—C5—H5	121.5	H10A—C10—H10B	109.5
C6—C5—H5	121.5	O5—C10—H10C	109.5
C1—C6—C5	119.8 (2)	H10A—C10—H10C	109.5
C1—C6—C7	122.8 (3)	H10B—C10—H10C	109.5
C5—C6—C7	117.4 (2)		
			
C10—O5—C2—C3	16.9 (5)	O4—C3—C4—O3	−0.4 (3)
C10—O5—C2—C1	−164.8 (3)	O3—C4—C5—C6	179.0 (3)
C6—C1—C2—O5	−177.1 (3)	C3—C4—C5—C6	2.0 (4)
Br1—C1—C2—O5	0.5 (4)	C2—C1—C6—C5	−0.9 (4)
C6—C1—C2—C3	1.3 (4)	Br1—C1—C6—C5	−178.4 (2)
Br1—C1—C2—C3	179.0 (2)	C2—C1—C6—C7	−179.2 (3)
O5—C2—C3—O4	−0.2 (5)	Br1—C1—C6—C7	3.3 (4)
C1—C2—C3—O4	−178.5 (3)	C4—C5—C6—C1	−0.8 (4)
O5—C2—C3—C4	178.2 (3)	C4—C5—C6—C7	177.6 (3)
C1—C2—C3—C4	−0.1 (4)	C8—O2—C7—O1	−1.4 (5)
C9—O4—C3—C2	−177.9 (3)	C8—O2—C7—C6	−179.1 (2)
C9—O4—C3—C4	3.6 (3)	C1—C6—C7—O1	34.9 (5)
C9—O3—C4—C5	179.7 (3)	C5—C6—C7—O1	−143.4 (3)
C9—O3—C4—C3	−3.0 (3)	C1—C6—C7—O2	−147.4 (3)
C2—C3—C4—C5	−1.6 (4)	C5—C6—C7—O2	34.2 (4)
O4—C3—C4—C5	177.0 (3)	C4—O3—C9—O4	5.2 (4)
C2—C3—C4—O3	−179.1 (3)	C3—O4—C9—O3	−5.5 (4)
